# An Oral Odyssey: Navigating the Complexity of Impacted Parapremolars and Paramolars in the Oral Landscape!

**DOI:** 10.7759/cureus.53056

**Published:** 2024-01-27

**Authors:** Mrudula Shinde, Pallavi Daigavane, Ranjit Kamble, Nishu Agarwal, Dhwani Suchak, Utkarsha S Chaudhari

**Affiliations:** 1 Orthodontics and Dentofacial Orthopaedics, Sharad Pawar Dental College and Hospital, Datta Meghe Institute of Higher Education and Research, Wardha, IND

**Keywords:** dental follicle, impacted, supernumerary teeth, parapremolar, paramolar

## Abstract

Supernumerary molars are an uncommon anomaly that can occur in the maxillofacial complex, referring to the presence of additional teeth in the dental arch. This condition is often associated with several rare disorders such as Gardner's syndrome, Cleidocranial dysplasia, Ehler-Danlos syndrome, and Down syndrome However, it is seldom observed in non-syndromic associations. They occur more frequently in the maxilla than in the mandible. This research represents a unique case study that explores unerupted paramolar and parapremolar follicles coexisting in both the maxillary and mandibular arches of a 17-year-old male patient. The discovery of these follicles was fortuitous, as the patient exhibited no symptoms during the initial orthopantomogram scan. Despite the absence of noticeable symptoms, vigilant monitoring and observation were employed over time. Subsequently, a substantial shift in the approach to management occurred with the integration of surgical and orthodontic methodologies, guided eruption strategies, and a collaborative, multidisciplinary effort.

## Introduction

Supernumerary teeth are extra tooth-like structures that can emerge alongside the normal dental pattern. These teeth can manifest in various ways within the oral cavity, such as solitary or numerous, impacted or erupted, and located on one or both sides of the jaw. It's important to note that the incidence of supernumerary teeth in permanent dentition ranges from 0.1% to 3.8% [1].

Studies conducted by Brook and Luten elucidate the prevalence of supernumerary teeth. Brook reported their presence in 2.1% of permanent dentitions and 0.8% of primary dentitions among the 2000 school children surveyed. Luten concentrated on supernumerary premolars in permanent dentition, observing that only 10% of all supernumerary cases comprised them, with a prevalence ranging from 0.075% to 0.26%. In primary and mixed dentitions, the lower incisors (50%) are followed by the mesiodens (36%), and the upper central incisors (11%) in decreasing sequence of frequency [1,2].

Supernumerary teeth are an oral anomaly that frequently manifests in various locations in the mouth, such as the midline of the maxilla, the palatal region of the upper incisors, the lower premolar area, and the distal regions around the upper and lower third molars. These additional dental structures can have a supplementary form that closely resembles conventional teeth, or they can appear primitive, with conoid, dysmorphic, or tubercular traits.

It is infrequent for individuals without genetic disorders to exhibit supernumerary teeth, occurring in only 1% of cases. However, genetic conditions such as Gardner's syndrome, Cleidocranial dysplasia, and Down syndrome are often accompanied by extra teeth. In this case, an asymptomatic 17-year-old male was inadvertently discovered to have unerupted paramolar and parapremolar in both his maxillary and mandibular arches during an orthopantomography. These findings accentuate the presence of several supernumerary teeth. This case report continues the clinical oral odyssey case series of multiple impacted supernumerary teeth of cleidocranial dysplasia [3].

According to previous research, the incidence of supernumerary molars varies from 0.001% to 0.57% [4]. However, certain studies have suggested that the prevalence of supernumerary molars exceeds 0.5%, or 2.1% [5]. Among the majority of studies carried out on the Turkish population, distomolars were observed to be more frequent than paramolars among supernumerary molars [6]. Conversely, paramolars were more common than distomolars in Cassetta et al.'s study on the Italian Caucasian population [7] and Kumar and Gopal's study on the Chennai population in India [8].

## Case presentation

**Figure 1:**
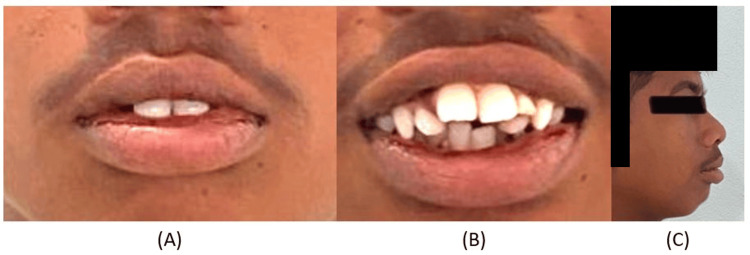
Pre-treatment extraoral photographs. (A) Frontal, (B) smiling, and (C) profile

A male patient aged 17 years presented to the Department of Orthodontics with concerns regarding the irregular and forward positioning of his teeth in the maxillary and mandibular anterior regions, and an over-retained deciduous tooth in the maxillary left region. The patient had a non-consonant smile, with a mesoproscopic face having a convex profile, and potentially competent lips (Figure 1).

**Figure 2:**
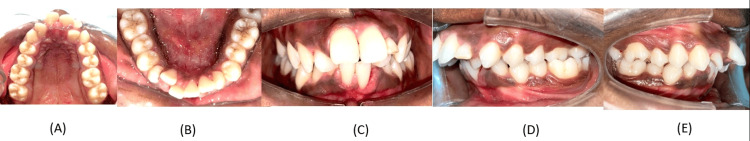
Pretreatment intra-oral photographs. (A) Maxillary arch, (B) mandibular arch, (C) anterior teeth in occlusion, (D) left molar in occlusion, and (E) right molar in occlusion

Upon intra-oral examination, it was found that all teeth, except for the third molars were present in both arches. The periodontal health of the patient seemed adequate, with a sufficient zone of attached gingiva. The molars had a class II relationship, and there was an overjet of 12mm (Figure 2).

**Figure 3:**
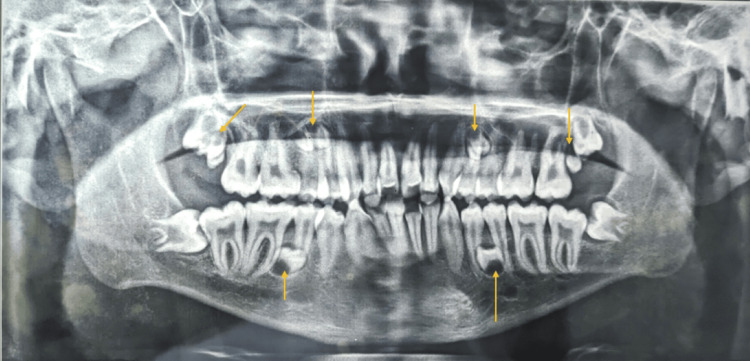
Pretreatment OPG (orthopantomogram) with parapremolar and paramolar in respective quadrants.

**Figure 4:**
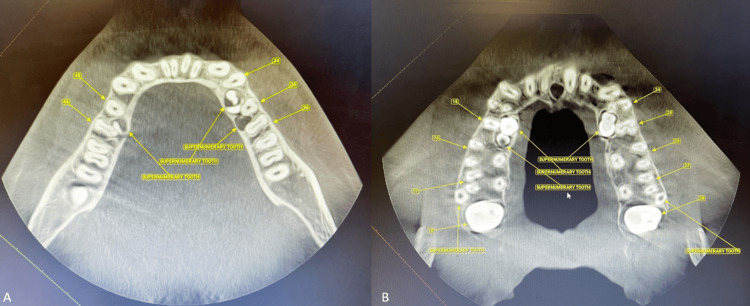
CBCT scan showing impacted teeth in respective arches. (A) Axial view - mandible, (B) Axial view - maxilla CBCT- Cone beam CT

The results of the functional examination indicated that the patient's speech, breathing, and swallowing functions were within normal limits. Moreover, the mandibular path of closure was assessed to be normal with no indication of deviation or temporomandibular disorder (TMD). Written and informed consent were obtained from the patient for the present case report. It was recommended that the patient undergo radiological examinations to determine the orientation of the root. After a panoramic examination (Figure 3), it was observed that the individual had impacted maxillary and mandibular parapremolars and paramolars in each of the maxillary and mandibular left and right quadrants. Furthermore, the maxillary and mandibular incisors were found to be proclined. Cone beam CT (CBCT) views of all quadrants also show the impacted supernumerary teeth (Figure 4).

Treatment plan

The therapeutic strategy was formulated to establish bilateral canine and molar relationships, ensuring appropriate occlusion, achieving space closure, and optimizing overjet and overbite parameters while ensuring coincident midlines. To attain these objectives, corrective measures are employed to initial alignment of teeth, alleviate crowding within both the maxillary and mandibular arches, and correct of proclination of the maxillary and mandibular incisors. The ultimate goal of this intervention is to produce a harmonious smile, enhance facial aesthetics, and achieve overall dental balance.

Within the framework of the therapeutic regimen, the over-retained supernumerary teeth located within both the mandibular and maxillary arches were systematically extracted after verifying the root development and assessing the condition of the permanent dentition. Given the complexities of the case, there was a requisite for a critical anchorage unit within both the upper and lower dental arches. The orthodontic approach commenced utilizing the pre-adjusted edgewise appliance (McLaughlin, Bennett and Trevisi (MBT) 0.022", Liberal Traders, New Delhi, India) slot for optimal management and alignment. An orthodontic fixed functional appliance was employed to rectify the convex profile and address the Class II molar relationship. After the completion of the requisite adjustments, meticulous finishing and detailing procedures were executed to ensure optimal outcomes.

## Discussion

Supernumerary teeth are morphologically normal or abnormal and develop from excess dental lamina in the jaws during tooth formation in intrauterine life. Supernumerary teeth can form due to unknown developmental defects or underlying genetic reasons, and early extraction is recommended. Mahto et al. reported the occurrence of posterior maxillary supernumerary teeth bilaterally [9]. Multiple impacted supernumerary teeth occur in Nicolaides-Baratsier syndrome along with severe mental impairment with delay in speech, seizures, short stature, brachydactyly, and sparse hair [10]. Cleidocranial dysplasia is characterized by multiple supernumerary teeth, multiple missing teeth, and a partial or complete absence of clavicle. Gardner's syndrome has multiple osteomas along with supernumerary teeth. Type I trichorhinophalangeal syndrome is an autosomal dominant disorder that includes supernumerary teeth in the maxillary molar region, long flat philtrum, protruding ears, thin maxillary vermilion border of the lip, a bulbous tip of the nose, skeletal anomalies with cone-shaped phalangeal epiphysis, and sparse scalp hair [11].

Supernumerary impacted teeth can have a significant impact on both the functionality and aesthetics of teeth. Adequate alignment within the dental arch is crucial when these teeth are found in the posterior region. Impactions such as paramolar, parapremolar, and distomolar are typically severe and require surgical intervention. A meticulous technique is used to reflect the buccal and palatal flaps while removing bone from the affected tooth. The successful outcome of these treatments depends upon the seamless coordination between orthodontists and oral surgeons, whose collective expertise delivers optimal results and a positive long-term prognosis. Surgical orthodontic techniques have proven to be successful in clinical settings. Established techniques for supernumerary tooth impaction include creating space in the dental arch and using surgical exposure and mechanical tension.

## Conclusions

Recognizing and addressing impacted supernumerary teeth promptly is crucial for maintaining optimal oral health within the patient's dental arches. The intricate nature of impaction necessitates a personalized treatment strategy for effective resolution. The synergistic efforts of orthodontic and surgical specialists, coupled with the sequential implementation of orthodontic and surgical phases, have revolutionized the management of impacted supernumerary teeth. This collaborative approach not only results in a remarkable improvement in aesthetics, functionality, and long-term oral well-being but also signifies a significant stride in the advancement of dental care. By prioritizing the identification and treatment of supernumerary teeth within the patient's dental arches, this integrated approach ensures a comprehensive and tailored solution for enhanced oral health outcomes.
